
JOURNAL INFORMATION
==============================
NLM Title Abbreviation: ScientificWorldJournal
Journal ID: ScientificWorldJournal
NLM Journal ID: 101131163
Journal ID: TSWJ

The Scientific World Journal

ISSN: 2356-6140
EISSN: 1537-744X
Publisher: Wiley

ARTICLE INFORMATION
==============================
PMCID: PMC6009348
PMID: 29973826
DOI: 10.1100/tsw.2002.51
Article ID: 484829
Article version: 1
Subjects: Short Report

Chemical Genetic Analysis of Protein Kinase Cascades

Alaimo Peter J.
Shogren-Knaak Michael A.
Shokat Kevan M. *
Department of Cellular and Molecular Pharmacology, University of California, San Francisco CA, USA
*Kevan M. Shokat: shokat@cmp.ucsf.edu
Publication date: 2002
Electronic publication date: 2002 Feb 5
Volume: 2
First page: 108
Last page: 110
Copyright: Copyright © 2002 Peter J. Alaimo et al.
Copyright year: 2002
License: This is an open access article distributed under the Creative Commons Attribution License, which permits unrestricted use, distribution, and reproduction in any medium, provided the original work is properly cited.
License URL: https://creativecommons.org/licenses/by/3.0/

==============================
Advances in Gene Technology: The Genome and Beyond –
Structural Biology for Medicine (Proceedings of the 2002 Miami
*Nature Biotechnology* Winter Symposium)
*TheScientificWorld* 2002, 2(S2), 108–110
ISSN 1532-2246; DOI 10.1100/tsw.2002.51

### CHEMICAL GENETIC ANALYSIS OF PROTEIN KINASE CASCADES

Peter J. Alaimo, Michael A. Shogren-Knaak, and Kevan M. Shokat
Department of Cellular and Molecular Pharmacology, University of California, San Francisco CA 94143-0450
shokat@cmp.ucsf.edu

**INTRODUCTION.** Renewed appreciation for the power of small organic molecules to address questions in cell biology has fueled an explosion of interest in chemical biology[1]. Using small molecules to perturb protein function has many advantages including: the effects of drugs are (1) rapid, potentially diffusion-limited, (2) often reversible due to metabolism/clearing, (3) tunable, allowing for graded phenotypes by varying concentration, and (4) conditional, since they can be introduced at any point in development.

Despite these advantages, the use of small molecules to probe cellular signaling has lagged behind that of genetic or biochemical methods. When trying to perturb biological function using small molecules the central problem is how to find small molecules that interact specifically with a desired protein target. Generating drug specificity is especially challenging when the protein target shares a high degree of homology with other proteins in the cell (such as protein kinases, GTPases, etc.). However, when using drugs as biological probes, one is not limited to modifying the small molecule. DNA cloning allows for modulation of protein structure by introducing point mutations or even entire protein domains. *Thus, it is possible to either start with a high-affinity, but nonspecific, inhibitor and confer specificity by site-directed mutagenesis, or to genetically introduce protein domains, which already possess high affinity and specificity.* Our lab has developed a combined chemical genetic method to specifically inhibit protein kinases[2]. Protein kinases form a large family and play a significant role in nearly all signaling pathways. The active site of protein kinases is well conserved and makes specific inhibition of a desired kinase challenging.

**METHODS.** In our approach, we use protein design to engineer a functionally silent yet structurally significant mutation into the active site of a kinase of interest (Figure). In our case this mutation is the replacement of a conserved bulky residue with glycine or alanine, thus creating a new pocket in the active site. Separately, a nonspecific kinase inhibitor of the wild-type enzyme has been chemically modified with substituents that specifically complement the mutation introduced into the active site. Importantly the new inhibitor analogs (1Na-PP1 [Figure] and 1NM-PP1) are designed to be unable to inhibit any wild-type kinases due to sterics.. Using this approach, several analogs of a pyrazolo[3,5-*d*]pyrimidine inhibitor have been found that inhibit engineered kinases with nanomolar $IC_{50}$s, but without significant inhibition of wild-type kinases.

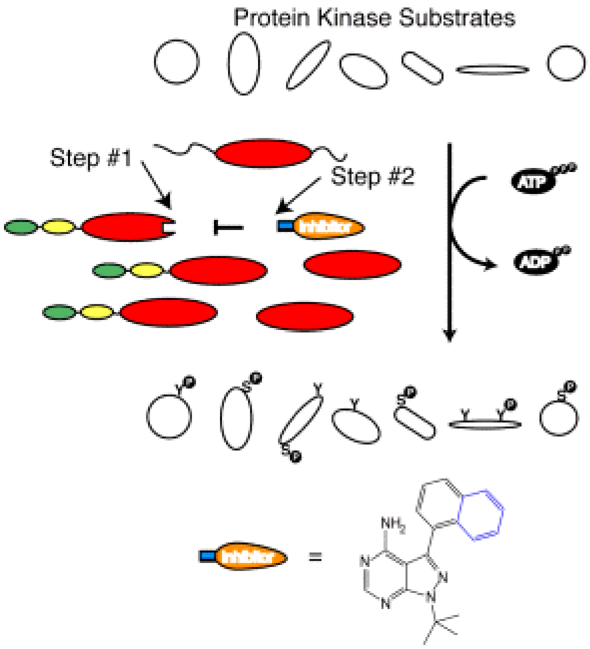

**FIGURE.** Additional color illustrations pertaining to this Report may be inspected by visiting (http://www.ucsf.edu/shokat/research.html).

**RESULTS.** This strategy has been used in *S. cerevisiae* to probe the role of the kinase Cdc28, which is the primary cyclin-dependent kinase (CDK) that controls the cell cycle in budding yeast[2]. Study of temperature-sensitive (TS) mutants of Cdc28 primarily indicated that the most critical role of this kinase was in the transition between the G1 and S phases of the cell cycle. Our lab in collaboration with the lab of David Morgan constructed yeast strains containing inhibitor-sensitive mutations in Cdc28, and tested them using our modified inhibitors. The expectation was that treatment of the strain carrying only an inhibitor-sensitive Cdc28 would show an identical G1/S transition arrest point. Surprisingly, however, it was found that drug-dependent arrest occurred at the G2/M transition in cells treated with 500 nM of 1-NM-PP1. Importantly, the discrepancy between genetic (TS) perturbation of cdc28 activity and chemical inhibition of cdc28 activity was not due to any "off-target" effects of the 1-NM-PP1 compound because this compound induced no toxicity or cell cycle arrest in wild-type yeast cells, which lacked the analog sensitive cdc28 allele. Thus, the difference in cellular phenotype must have been the result of a fundamental difference in how cdc28 function was altered by the two approaches.

Typically, temperature-sensitive mutants unfold at elevated temperature (the so-called nonpermissive temperature) thus resulting in a total loss of protein function. Since protein kinases possess both catalytic function (phosphorylation activity) and protein-protein association functions (so-called scaffolding functions), a temperature sensitive phenotype can be thought of as a complete loss of function phenotype. On the other hand, the chemical inhibitor blocks only one of the possible functions of the protein kinase, it's catalytic phosphorylation function, since it is an ATP competitive binder. Thus, the discrepancy between chemical inhibition of cdc28 and temperature-induced unfolding can likely be ascribed to the importance of scaffolding functions of cdc28 at different points of the cell cycle. Furthermore, the G2/M arrest point induced by chemical inhibition of cdc28 agreed with a well-established relationship between cdc28 catalytic activity and cell cycle progression. The catalytic activity of cdc28 increases steadily from G1-S-G2-M phases of the cell cycle, peaking at M phase. One view of the cell cycle is that progression through the cell cycle requires increasing cdc28 activity for cell cycle progression. Our finding that inhibition of cdc28 activity resulted in a G2/M arrest point agrees well with this model. Furthermore, the model predicts that at higher inhibitor concentration, an earlier cell cycle arrest should arise. This was tested by increasing the dosage of 1-NM-PP1 from 500 nM (G2/M arrest) to 5000nM—and in fact a clear new phenotype was revealed—a G1/S arrest, just as the model predicted. The ability of this chemical genetic approach to identify new functions of the well-studied cell cycle kinase, Cdc28, highlights the advantages of using chemical genetics as a complement to traditional genetics.

**CONCLUSION.** Approaches that combine chemistry and genetics offer a means of generating small molecules that act rapidly and reversibly, yet offer the portability and specificity of genetics. We expect that with the current interest in proteomics and genomics, these techniques will continue to be important for understanding complex signaling networks, and related methods will continue to be developed.

**REFERENCES**

1. Alaimo, P.J., Shogren-Knaak, M.A., and Shokat, K.M. (2001) Curr. Opin. Chem. Biol. 5, 360–367.
2. Bishop, A.C. et al. (2000) Nature 407, 395–401.
